# Evaluation of Safety, Immunogenicity and Cross-Reactive Immunity of OVX836, a Nucleoprotein-Based Universal Influenza Vaccine, in Older Adults

**DOI:** 10.3390/vaccines12121391

**Published:** 2024-12-11

**Authors:** Bart Jacobs, Isabel Leroux-Roels, Jacques Bruhwyler, Nicola Groth, Gwenn Waerlop, Yorick Janssens, Jessika Tourneur, Fien De Boever, Azhar Alhatemi, Philippe Moris, Alexandre Le Vert, Geert Leroux-Roels, Florence Nicolas

**Affiliations:** 1 Center for Vaccinology (CEVAC), 10 Corneel Heymanslaan, 9000 Ghent, Belgium; bart.jacobs2@uzgent.be (B.J.); isabel.lerouxroels@uzgent.be (I.L.-R.); gwenn.waerlop@ugent.be (G.W.); yorick.janssens@ugent.be (Y.J.); fien.deboever@uzgent.be (F.D.B.); azhar.alhatemi@uzgent.be (A.A.); geert.lerouxroels@ugent.be (G.L.-R.); 2 Osivax, 70 Rue Saint-Jean-de-Dieu, 69007 Lyon, France; ngroth@osivax.com (N.G.); jtourneur@osivax.com (J.T.); pmoris@osivax.com (P.M.); alevert@osivax.com (A.L.V.); fnicolas@osivax.com (F.N.)

**Keywords:** influenza, universal vaccine, Phase 2a, healthy participants, older adults, cross-reactivity, safety, immunogenicity

## Abstract

**Background/Objectives:** In a Phase 2a, double-blind, placebo-controlled study including healthy participants aged 18–55 years, OVX836, a nucleoprotein (NP)-based candidate vaccine, previously showed a good safety profile, a robust immune response (both humoral and cellular) and a preliminary signal of protection (VE = 84%) against PCR-confirmed symptomatic influenza after a single intramuscular dose of 180 µg, 300 µg or 480 µg. **Methods**: Using the same methodology, we confirmed the good safety and strong immunogenicity of OVX836 at the same doses in older adults (≥65 years), a key target population for influenza vaccination. **Results**: Significant humoral (anti-NP IgG) and cellular (interferon gamma (IFNγ) spot-forming cells per million peripheral blood mononuclear cells and specific CD4^+^ IFNγ^+^ T-cells) immune responses were observed at the three dose levels, without clear dose–response relationship. T-cell responses were shown to be highly cross-reactive against various influenza A strains, both seasonal and highly pathogenic avian strains. We also evaluated the effect of sex (stronger immune response in females) and age (stronger immune response in young adults) on the immune response to OVX836 after adjustment based on the pre-vaccination immune status. **Conclusions**: The results obtained with OVX836 lay the groundwork for a future placebo-controlled, field proof of concept efficacy Phase 2b trial.

## 1. Introduction

Preventing influenza remains an important challenge for the medical community, with an estimated 290,000–650,000 deaths annually worldwide, based on data from 1999–2015 [1]. Currently available quadrivalent influenza vaccines, inducing antibody responses and targeting virus surface glycoproteins (haemagglutinin (HA) and neuraminidase), have relatively limited and highly variable efficacy across seasons, particularly among individuals aged 65 years and older [2,3,4,5]. Cell-mediated immune (CMI) responses, in particular the stimulation of CD4^+^ and CD8^+^ T-cells, could also contribute to the protection against influenza infection [2,6,7,8,9,10,11].

The OVX836 vaccine candidate (Osivax, Lyon, France) consists of a recombinant protein, including the nucleoprotein (NP) of the influenza virus (strain A/WSN/1933[H1N1]), combined with OVX313 (oligoDOM^®^), a self-assembling nanoparticle technology (Osivax, Lyon, France). This protein spontaneously assembles into nanoparticles, which are positively charged and consist of seven copies of the NP fused to OVX313 [12,13]. The influenza NP is critical for the virus replication process and has a low mutation rate, making it an attractive target for the development of a universal influenza vaccine [14,15,16].

In a Phase 2a, double-blind, placebo-controlled study, the immunogenicity and safety of a single intramuscular administration of OVX836 have been assessed at three dose levels (180 µg, 300 µg and 480 μg) in healthy subjects aged 18–55 years and in older adults aged 65 years and above. The results from the younger adult cohort have recently been published [17]. In this part of the study, OVX836 induced robust humoral anti-NP immunoglobulin G (IgG) response, as well as CMI responses, translating into an increase in the number of NP-specific interferon gamma (IFNγ) spot-forming cells (SFCs) per million peripheral blood mononuclear cells (PBMCs) and in the percentage of polyfunctional NP-specific CD4^+^ T-cells. A CD8^+^ T-cell response was elicited at the 300 µg and 480 µg dose levels. OVX836 demonstrated a favorable safety profile up to 480 µg, with no dose-dependent increase in reactogenicity and no indication of reaching the maximum tolerated dose. A vaccine efficacy signal was observed in an epidemiological context of H3N2 circulation, with an observed vaccine efficacy (VE) of 84% (95% confidence interval = 17–97%).

The aims of this paper are to present the safety and immunogenicity results from the second part of the study conducted in older adults aged 65 years and over to assess the cross-reactivity of OVX836-induced CMI against the NP from heterologous influenza virus A and B strains both in older and younger participants and to evaluate the influence of baseline (pre-vaccination) immunological readouts, age and sex on the immune response to OVX836.

## 2. Methods

The study methods (ClinicalTrials.gov NCT05060887 and EudraCT 2021-002535-39) have been thoroughly described in a previous publication [17]. In brief, the study was conducted in a single center (Centre for Vaccinology (CEVAC), Ghent University and University Hospital, Ghent, Belgium), in accordance with good clinical practice and was approved by the Ethics Committee of the Ghent University Hospital and by the Belgian Federal Agency for Medicines and Health Products (FAMHP). All participants provided written informed consent prior to enrolment. In the second part of the study, carried out after the 2021–2022 influenza season, 100 participants aged 65 years and over were randomized into four equal groups of 25 participants, (1:1:1:1 ratio) to receive either OVX836 at 180 µg, 300 µg or 480 µg or a placebo (saline solution) administered into the deltoid muscle.

The list of eligibility criteria, as listed in the clinical study protocol, can be found in Supplementary S1.

Vaccine reactogenicity (consisting of local (injection site pain, redness and swelling) and systemic (fatigue, headache, arthralgia, malaise, myalgia and fever) solicited adverse events (AEs)) was recorded in a paper diary for 7 days post-vaccination. All the other AEs and serious AEs (SAEs) were reported and monitored for 28 and 180 days post-vaccination, respectively. All participants attended visits at the investigators’ site on Day 1, Day 8 and Day 29 post-injection. 

Blood samples were collected on Day 1 (serum and PBMCs), Day 8 (serum and PBMCs) and Day 29 (serum only). All samples were frozen and all time points were assessed simultaneously. An electronic case report form was used for the data collection.

The two primary endpoints were: (1) the change in NP-specific IFNγ SFC frequencies in the PBMCs, measured by IFNγ ELISpot on Day 8 versus pre-vaccination baseline (Day 1) at the three dose levels of OVX836; and (2) the number and percentage of participants reporting solicited AEs, unsolicited AEs and/or SAEs in the OVX836 groups compared to placebo.

The secondary endpoints included: (1) the percentage of NP-specific CD4^+^ and CD8^+^ T-cells expressing interleukin 2 (IL2), tumor necrosis factor alpha (TNFα) and/or IFNγ (and all possible combinations of these cytokines) upon in vitro stimulation, measured by flow cytometry; and (2) the geometric mean titers (GMTs) of anti-NP IgG, measured by ELISA in serum.

As a post hoc exploratory endpoint, selected PBMC samples from young and older participants were stimulated with NP peptide pools from H1N1-A/WSN/1933 (homologous at >90% to OVX836) and heterologous strains, including pH1N1-A/California/04/2009, H3N2-A/Kansas/14/2017, H5N1-A/Indonesia/5/2005 and B/Colorado/06/2017. The IFNγ ELISpot assay was used to enumerate NP-specific precursor cells capable of producing IFNγ upon stimulation with the relevant antigens, allowing for a comparison of responses. Detailed immunoassay methodologies are provided in Supplementary S2.

The second part of the study involving older participants was not powered to test any statistical hypotheses. The statistical methods used were essentially descriptive, however, some post hoc inferential analyses were performed without correction for multiplicity and should be regarded as exploratory. Details of the statistical methodologies are provided in Supplementary S3.

## 3. Results

### 3.1. Study Population Demographics and Baseline Characteristics

**Figure 1:**
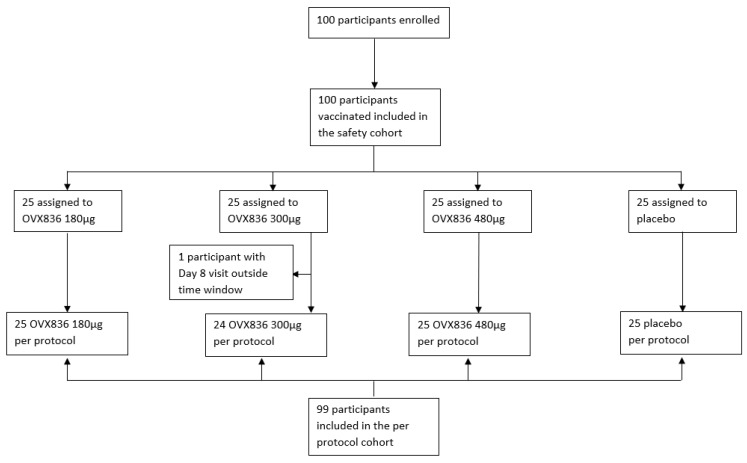
CONSORT diagram of the OVX836-003 study (Part II consisting of participants aged 65 years and over).

Vaccinations were performed after the influenza season, between 2 May and 21 June 2022, as the Ethics Committee did not approve the administration of a placebo in older participants during a period of influenza circulation. The last participant’s visit (Day 180) took place on 7 December 2022. A total of 100 participants were enrolled and received either the study vaccine or placebo, with 25 participants assigned to each treatment group. The per-protocol cohort consisted of 99 participants (24 in the OVX836 300 µg group and 25 in the other groups). All 100 participants completed the study (Figure 1).

All participants were White Caucasians, with a mean age of 71.0 ± 5.0 years (range: 65–85 years). The sex ratio was balanced, with 49% females. The baseline characteristics were comparable across the four treatment groups. Eighty percent of the participants had been vaccinated against influenza during the autumn 2021, prior to their inclusion in the study (Table 1).

### 3.2. Reactogenicity and Safety

Solicited local reactions (essentially mild to moderate injection site pain) were more frequent in the three OVX836 groups compared to the placebo group, without significant differences between the three OVX836 dose levels. The percentage of participants reporting systemic solicited symptoms (none severe) and unsolicited AEs were similar across the three OVX836 groups. There were only two severe AEs that were not considered related to the vaccine or placebo: one in the OVX836 180 µg group (nephrolithiasis) and one in the placebo group (cataract). Five SAEs were reported: three in the OVX836 180 µg group (flank pain, syncope and cystocele), one in the OVX836 300 µg group (head injury) and one in the OVX836 480 µg group (non-small cell lung cancer). None of these were deemed related to the vaccine (Table 2).

### 3.3. Cell-Mediated Immune Response

**Figure 2:**
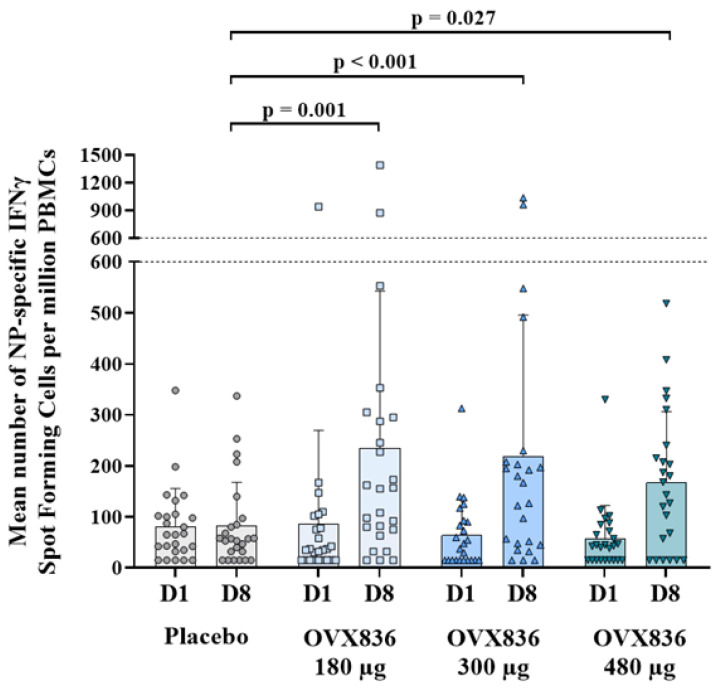
Number of nucleoprotein (NP)-specific IFNγ spot-forming cells (SFCs) per million peripheral blood mononuclear cells (PBMCs) after a single intramuscular administration of OVX836 (180 μg, 300 μg or 480 μg) or placebo. Data are mean (standard deviation) and individual values on Day 1 (before vaccination) and Day 8 (7 days following vaccination). The three vaccine groups significantly differed from the placebo: *p* = 0.001 for OVX836 180 µg, *p* < 0.001 for OVX836 300 µg and *p* = 0.027 for OVX836 480 µg. The differences between the OVX836 groups themselves were not statistically significant (*p* = 1.000).

OVX836 increased the frequency of NP-specific IFNγ SFCs in PBMCs, as measured by ELISpot on Day 8. Statistically significant (*p* = 0.001 for OVX836 180 µg, *p* < 0.001 for OVX836 300 µg and *p* = 0.027 for OVX836 480 µg) increases were observed for all three OVX836 dose levels compared to placebo, with no significant differences among the OVX836 dose levels themselves (*p* = 1.000). There were no differences between groups at baseline (Day 1), and no responses were recorded in the placebo group on Day 8 (Figure 2 and Supplementary S4).

**Figure 3:**
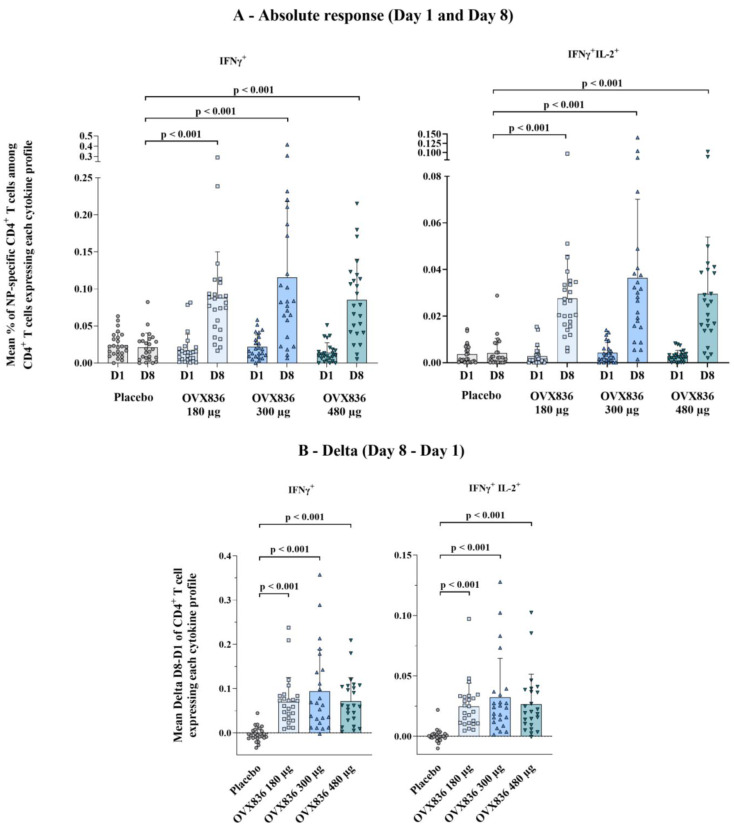
Percentage of nucleoprotein (NP)-specific CD4^+^ T-cells expressing at least interferon gamma (IFNγ) or IFNγ and interleukin-2 (IL-2) following a single intramuscular dose of OVX836 (180 µg, 300 µg or 480 µg) or placebo. Panel (**A**) shows the mean (standard deviation) and individual percentage of NP-specific CD4^+^ T-cells expressing at least IFNγ or IFNγ and IL-2 on Day 1 (prior to vaccination) and Day 8 (7 days following vaccination). Panel (**B**) shows the absolute differences (delta) between Day 8 and Day 1. The three vaccine groups significantly differed from the placebo (*p* < 0.001). The differences between the OVX836 groups themselves were not statistically significant (*p* = 1.000).

In the three OVX836 groups, statistically significant increases (*p* < 0.001) were observed in the percentage of NP-specific CD4^+^ T-cells identified as expressing at least IFNγ or a combination of IFNγ and IL2 compared to pre-vaccination values (Figure 3 and Supplementary S4), with no pairwise statistically significant differences between dose levels (*p* = 1.000). No changes were noted in the placebo group between Day 8 and Day 1. 

No significant increases in CD8^+^ T-cells were detected at any OVX836 dose level (Supplementary S4).

### 3.4. Humoral Immune Response

**Figure 4:**
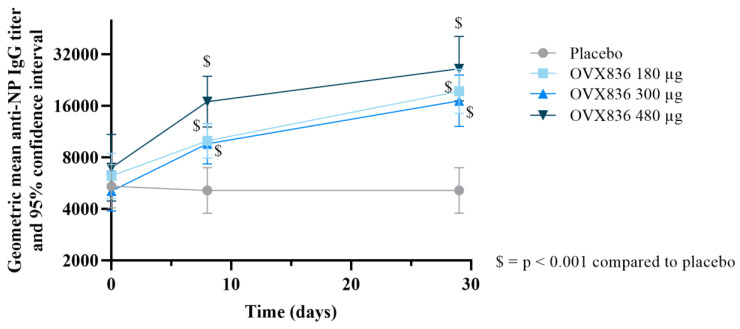
Anti-nucleoprotein (NP)-specific immunoglobulin G (IgG) titers determined at baseline (Day 1—pre-vaccination), Day 8 (7 days post-vaccination) and Day 29 (28 days post-vaccination) in the placebo and OVX836 vaccine groups (180 μg, 300 μg and 480 μg). Data are geometric mean titers ± 95% confidence intervals. At both post-vaccination timepoints (Day 8 and Day 29), GMTs in the three OVX836 dose groups were significantly higher than those in the placebo group (*p* < 0.001). The differences between the three OVX836 dose levels were not statistically significant (*p* > 0.05).

OVX836 induced a strong humoral immune response (Figure 4 and Supplementary S4). In all three OVX836 groups, geometric mean titers (GMTs) showed a statistically significant increase (*p* < 0.001) on both Day 8 and Day 29 compared to pre-vaccination levels (Day 1). While there was a trend indicating a dose-dependent effect, the differences between the OVX836 groups were not statistically significant (*p* > 0.05) at either time point (Day 8 or Day 29). No changes in anti-NP IgG GMTs were observed in the placebo group. On Day 29, the percentages of participants with a 4-fold increase in anti-NP IgG titers were 48%, 54% and 68% in the OVX836 180 µg, 300 µg and 480 µg groups, respectively.

In some subjects of the OVX836 vaccine groups, a moderate increase in anti-OVX313 IgG titers was measured. Although OVX313 is derived from an avian sequence of C4BP, no cross-reactivity with the human C4BP (hC4BP) oligomerization domain was detected.

### 3.5. Cross-Reactive Immunity

Post hoc exploratory analyses were conducted to assess the reactivity of the cellular immune response induced by OVX836 against the NPs from heterologous influenza virus A and B strains. This evaluation included 35 participants aged 18–55 years (26 females and 9 males; 20 participants in the OVX836 300 µg showing Day 8/Day 1 ratio ≥ 2.5 in terms of IFNγ ELISpot response and 15 in the OVX836 480 µg group, randomly selected) and 24 participants aged 65 years and older (18 females and 6 males; 8 participants per OVX836 dose group showing Day 8/Day 1 ratio ≥ 2.0 in terms of IFNγ ELISpot response).

**Figure 5:**
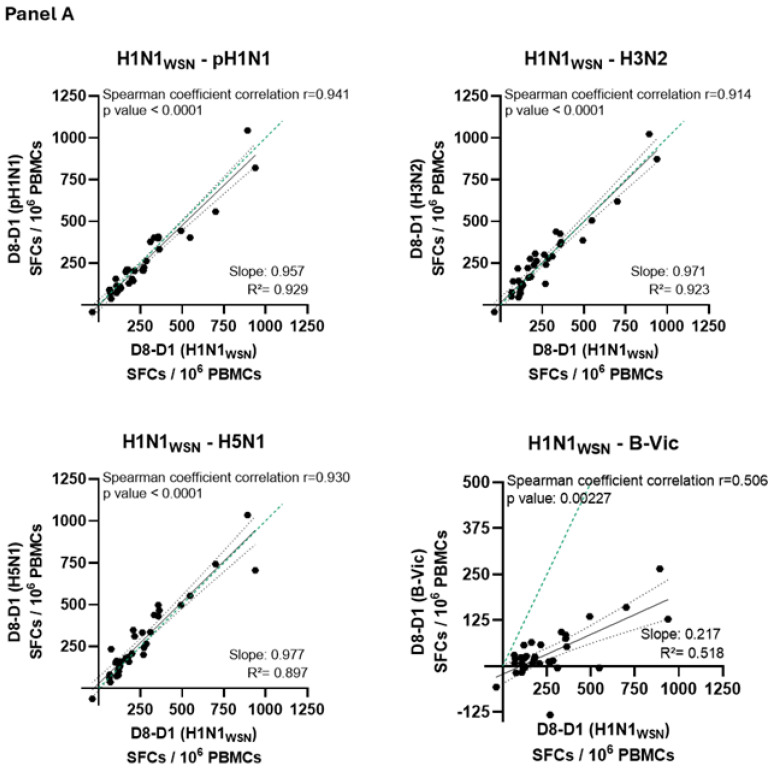

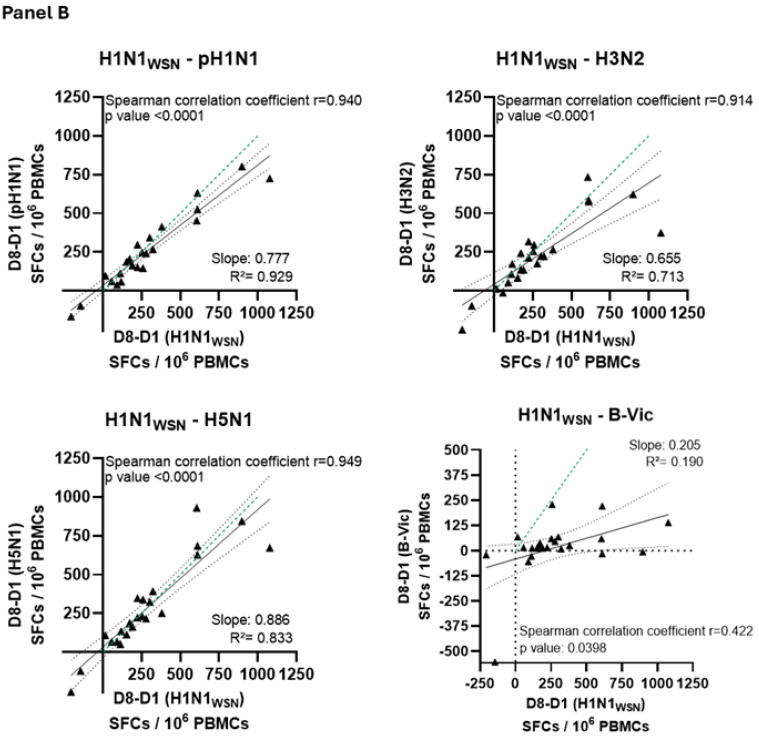
Correlation of the vaccine-induced interferon gamma (IFNγ) ELISpot responses (difference (delta) between Day 8 and Day 1) specific to the homologous nucleoprotein (NP) (H1N1-A/WSN 1933) versus the IFNγ responses (difference (delta) between Day 8 and Day 1) to NPs from heterologous influenza A strains (pH1N1-A/California, H3N2-A/Kansas and H5N1-A/Indonesia) and influenza B strain (B-Victoria). Panel (**A**) shows participants aged 18–55 years, while Panel (**B**) shows participants aged 65 years and older. The slope of the regression line is indicated in each figure (dotted green line: regression with slope = 1).

Figure 5 illustrates the magnitude of the IFNγ ELISpot responses elicited following stimulation with NP from H1N1-A/WSN/1933 (homologous to the vaccine) and NP from heterologous strains, including H1N1-A/California/04/2009, H3N2-A/Kansas/14/2017 H5N1-A/Indonesia/5/2005 and Victoria B/Colorado/06/2017.

A highly significant Spearman’s correlation coefficient (r ≥ 0.91, *p* < 0.0001) was observed between the response to OVX836 NP and various heterologous influenza A strain NPs (including H5N1) in both age groups. Regression slopes were ≥0.96 in participants aged 18–55 years and ≥0.66 in participants aged 65 and older, confirming the robust cross-reactivity of the immune response elicited by OVX836 vaccination across different influenza A strains. Additionally, a lower but statistically significant Spearman’s correlation coefficient was noted for the influenza B (Victoria) strain (r = 0.50, *p* < 0.01 in participants aged 18–55 years; r = 0.42, *p* < 0.05 in participants aged 65 years or older) with a modest regression slope around 0.20. This slope was significantly different from zero (*p* < 0.05) in both age cohorts, indicating cross-reactivity to influenza B as well, albeit to a lesser degree.

### 3.6. Parameters Influencing the Response to OVX836 Vaccination

The two age cohorts (18–55 years [17] and ≥65 years) were similar in terms of weight, body mass index, ethnicity/race and smoking status. However, they differed in the female/male ratios, with a ratio of 2.61 in the 18–55 years cohort and 0.98 in the ≥65 years cohort. Additionally, notable differences were observed between the two groups regarding baseline (pre-vaccination) immunological readouts (Supplementary S5). Overall, baseline CMI readouts were significantly higher (*p* < 0.01) in the 18–55 years cohort compared to the ≥65 years cohort, while baseline anti-NP IgG titers showed the opposite trend (*p* < 0.0001). Further statistical analyses were performed to evaluate the effects of different parameters (baseline, age and sex) on the immune response to OVX836.

#### 3.6.1. Influence of Pre-Vaccination Immunological Values on the Immune Response to OVX836

**Figure 6:**
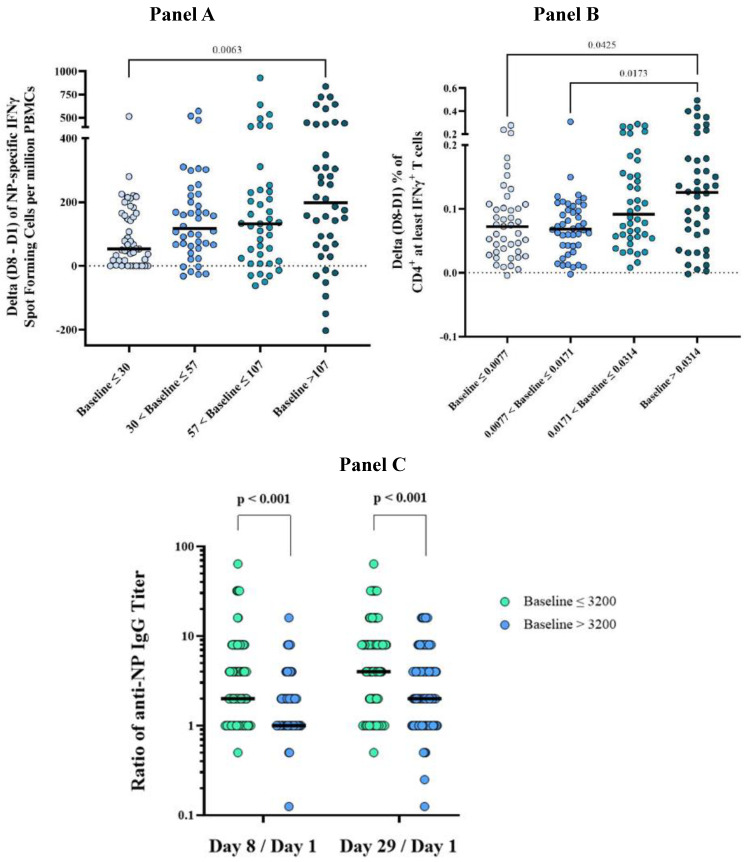
Influence of the baseline (pre-vaccination) immunological status on the response to OVX836 (180 µg, 300 µg and 480 µg). Panel (**A**) shows the differences between Day 8 and Day 1 (delta) in nucleoprotein (NP)-specific interferon gamma (IFNγ) spot-forming cells (SFCs) per million peripheral blood mononuclear cells (PBMCs). Panel (**B**) shows the differences between Day 8 and Day 1 (delta) in the percentage of CD4^+^ T-cells producing at least IFNγ. Panel (**C**) shows the geometric mean ratio (GMR) of anti-NP IgG titers (Day 8/Day 1 and Day 29/Day 1). Results are displayed as means with individual values for all participants, divided into four quartiles based on baseline ELISpot and CD4^+^ T-cell values, and as GMR with individual values based on the median baseline titer for anti-NP IgG.

It was observed that the baseline (pre-vaccination, Day 1) immunological status influenced the immune response to OVX836 (Figure 6). In the merged age cohorts, higher baseline values were associated with increased CMI response as measured by the differences in NP-specific IFNγ SFCs per million PBMCs between Day 8 and Day 1 (*p* = 0.006 for comparisons between quartiles 1 and 4) and an increase in the frequencies of NP-specific CD4^+^ (but not CD8^+^) T-cells expressing at least IFNγ (*p* < 0.05 for comparisons between quartiles 1 or 2 and quartile 4). 

Conversely, significantly lower anti-NP IgG geometric mean ratios (Day 8/Day 1 and Day 29/Day 1) were observed when baseline titers were higher.

#### 3.6.2. Effect of OVX836 Dose Levels, Sex and Age on the Immune Response to OVX836

Exploratory analyses were conducted to compare the immunological responses to the three dose levels of OVX836 (180 µg, 300 µg and 480 µg), accounting for differences in sex ratio between the younger and older cohorts. This was performed using analysis of covariance (ANCOVA) models, adjusted for baseline values.

After adjustment for baseline values, no significant global effects (*p* > 0.05) of vaccine dose, age or sex on CMI responses (measured by NP-specific IFNγ SFCs per 10^6^ PBMCs and percentage of CD4^+^ T-cells expressing IFNγ) were observed. However, younger participants (aged 18–55 years) tended to show higher responses compared to older participants (aged ≥ 65 years), as they started from higher baseline values (Supplementary S6.1). The dose–response relationship appeared more pronounced in young females compared to older females (Supplementary S6.2) or males (Supplementary S6.3).

Similarly, after baseline adjustment, the effect of the OVX836 dose on the Day 29/Day 1 anti-NP IgG geometric mean ratio (GMR) was not statistically significant (*p* > 0.05). However, when comparing age groups, younger participants (18–55 years) with lower baseline titers had significantly higher GMRs compared to older participants (*p* = 0.014) (Supplementary S7).

Due to the absence of a consistent and statistically significant dose–response relationship, the three OVX836 dose levels were pooled to increase sample size, allowing for a better assessment of the effects of age and sex on immunological responses, using the same ANCOVA models.

Overall, both CMI and humoral responses to OVX836 tended to be lower in older compared to younger participants and higher in females compared to males (Supplementary S8). While trends were noted in NP-specific IFNγ SFCs per 10^6^ PBMCs, these were not statistically significant (*p* > 0.05). For CD4^+^ T-cells, the effect of sex was statistically significant (*p* = 0.041), but the effect of age was not (*p* > 0.05). For anti-NP IgG Day 29/Day 1 GMRs, both sex (*p* = 0.025) and age (*p* = 0.004) had statistically significant effects.

## 4. Discussion

The good safety profile and robust immune response (both humoral and cellular) of OVX836, an NP-based universal influenza vaccine, across a dose range of 180 µg to 480 µg (administered as a single IM injection), has been previously demonstrated in participants aged 18–55 years [17,18]. In this paper, we present, for the first time, results of OVX836 vaccination in participants aged 65 years and older, a critical target population for influenza vaccination. The safety and immunogenicity of OVX836 in this population were confirmed, with statistically and clinically significant humoral and cellular immune responses observed at all three dose levels, though no clear dose–response relationship was established.

Interestingly, OVX836-induced NP-specific T-cell responses were shown to be highly cross-reactive against various influenza A strains, including both seasonal and one highly pathogenic avian strain. The cross-reactivity was consistent across younger and older participants, likely due to the high sequence similarity (95%) observed among the NP proteins of influenza A strains. In contrast, significantly lower cross-reactivity was observed for the influenza B strain which is consistent with the lower sequence homology between NP proteins of influenza A and B strains (50–60%). If vaccine efficacy is demonstrated in future field efficacy trials against seasonal influenza A strains, OVX836 could be a valuable tool in combating a potential influenza A pandemic. For seasonal influenza protection, OVX836 may require adaptation to include an influenza B NP or be administered alongside a traditional HA-based inactivated seasonal influenza vaccine. Nonetheless, these human cross-reactivity results are promising, positioning OVX836 as a potential candidate in the pursuit of a truly universal influenza vaccine [19].

Differences between the two age groups were observed in terms of baseline (pre-vaccination) immunological values. Baseline NP-specific CMI values (NP-specific IFNγ SFCs per 10^6^ PBMCs and frequency of NP-specific CD4^+^ T-cells identified as expressing IFNγ or at least one cytokine among IFNγ, IL-2 and TNFα) were significantly higher (*p* < 0.01) in the younger cohort (18–55 years) compared to the older cohort (≥65 years), whereas the opposite was true for baseline anti-NP IgG titers, which were significantly higher (*p* < 0.0001) in the older cohort.

The higher levels of circulating anti-NP IgG in older participants may be explained by their more frequent exposure to NP through natural influenza infections over time as compared to younger participants. However, this trend was not observed for NP-specific memory T-cells, likely because these cells are not prevalent in circulation, as they remain tissue-resident.

Baseline immunological status had a statistically significant impact on the immune response to OVX836 across all measured immunogenicity endpoints (*p* < 0.01). Higher baseline anti-NP IgG titers were associated with lower Day 8/Day 1 or Day 29/Day 1 ratios after OVX836 vaccination, a finding consistent with the phenomenon of antibody feedback inhibition, as seen with other vaccines, including the hemagglutination inhibition response following seasonal HA-based vaccinations [20,21]. Conversely, higher baseline CMI parameters were associated with greater post-vaccination increases in CMI responses (Day 8 versus Day 1), possibly reflecting the role of pre-existing memory T-cells in mounting a strong response to OVX836. 

After pooling of the OVX836 dose levels and adjusting for baseline values using ANCOVA models, trends were observed suggesting stronger immunological responses in females compared to males and in younger participants (18–55 years) compared to older participants (≥65 years).

A recent meta-analysis of randomized controlled trials confirmed that sex has a significant impact on the immunogenicity and efficacy of seasonal influenza vaccines [22]. Females, particularly those of reproductive age, tend to mount stronger immune responses, both innate and adaptive [23,24,25,26].

The impact of aging on the immune system is a complex phenomenon that continues to be investigated by many experts worldwide [27]. Several studies have shown that immune responses to vaccines are often less effective in older adults [28,29], a physiological decline known as immunosenescence [27,30,31]. Recent data suggest that aging causes a contraction in the pool of naive CD8^+^ T-cells due to reduced thymic output, while the pool of naive CD4^+^ T-cells is somewhat maintained through robust homeostatic proliferation [32]. In our study, immune responses, both cellular and humoral, to OVX836 were generally of lower amplitude in the older participants. Although the sample size is too small to draw definitive conclusions, it appears that the response to OVX836, while slightly diminished, is not necessarily impaired in older participants. 

The role of NP CD4+ and CD8+ T-cells in clearing the influenza infection in both human and animal models is largely supported by previous published works [2,6,7,8,9,10,33,34]. The situation is less clear about the role of anti-NP IgG. Anti-NP antibodies are not neutralizing as NP is an internal antigen of the influenza virus. These antibodies may nevertheless play a role in viral clearance through antibody-dependent cellular cytotoxicity (ADCC)/antibody-dependent phagocytosis [35,36] or play a role in resolving the infection through other mechanisms of action [37,38,39,40]. However, anti-NP IgG generated after OVX836 vaccination in mice was not protective in vivo when passively transferred into a murine influenza challenge [41], whereas other authors were able to demonstrate the protective effect of NP IgG in this animal model [42,43]. Overall, the role of anti-NP IgG in the protection against influenza in animal models and in humans still remains controversial and needs to be further investigated.

The interpretation of the study findings and their generalizability are limited by the small sample size. Additionally, borderline *p*-values should be interpreted cautiously, as multiple endpoints and analyses were not accounted for. In the absence of a proven correlate of protection, the efficacy of OVX836 will need to be demonstrated in larger field efficacy trials and the respective role of NP cellular and humoral responses in this protection will have to be further delineated. 

## 5. Conclusions

The study confirms that OVX836 is a safe and well-tolerated candidate vaccine, eliciting both humoral (anti-NP IgG) and cellular (T-cell) immune responses in both young and older adults. Although the sample size was too small to draw definitive conclusions, it appears that the response to OVX836, while slightly diminished, is not necessarily impaired in older participants, constituting the target population. Interestingly, OVX836 T-cell responses were highly cross-reactive, both in young and older subjects, against various influenza A strains, including both seasonal and one highly pathogenic avian strain. The data generated on OVX836 until today lay the groundwork for a future placebo-controlled, field efficacy Phase 2b trial of OVX836, particularly in the context of pandemic preparedness.

## Figures and Tables

**Table 1:** Demographics and other baseline characteristics of the study participants, overall and in the four treatment groups (OVX836 180 µg, 300 µg, 480 µg and placebo). Data are mean ± standard deviation for continuous variables and number (%) of participants for categorical variables.

	Placebo	OVX836 180 µg	OVX836 300 µg	OVX836 480 µg	All Participants
	N = 25	N = 25	N = 25	N = 25	N = 100
Age (year)	70.9 ± 5.6	70.4 ± 4.6	71.0 ± 4.1	71.8 ± 5.8	71.0 ± 5.0
Weight (kg)	75.9 ± 15.1	73.3 ± 13.7	75.2 ± 14.0	73.4 ± 13.5	74.4 ± 13.9
Height (cm)	172 ± 9	171 ± 8	167 ± 9	167 ± 8	169 ± 9
BMI (kg/m^2^)	25.3 ± 3.2	25.0 ± 2.9	26.9 ± 3.3	26.3 ± 3.5	25.9 ± 3.3
Sex (female)	10 (40.0)	12 (48.0)	14 (56.0)	13 (52.0)	49 (49.0)
Race (White)	25 (100.0)	25 (100.0)	25 (100.0)	25 (100.0)	100 (100.0)
Smoking status					
*Non-smoker*	9 (36.0)	16 (64.0)	16 (64.0)	14 (56.0)	55 (55.0)
*Current smoker*	2 (8.0)	3 (12.0)	2 (8.0)	1 (4.0)	8 (8.0)
*Former smoker*	14 (56.0)	6 (24.0)	7 (28.0)	10 (40.0)	37 (37.0)
Influenza vaccination (season 2021–2022)	20 (80.0)	19 (76.0)	21 (84.0)	20 (80.0)	80 (80.0)

**Table 2:** Number and percentage of subjects reporting solicited local and systemic adverse events (AEs; all grades and severe grade) during the 7-day period post-vaccination, and unsolicited AEs (overall, severe and considered related to the investigational product (IP) by the investigator) during the 28-day period post-vaccination, in the placebo and OVX836 groups.

	Intensity	OVX836 180 µg (N = 25)	OVX836 300 µg (N = 25)	OVX836 480 µg (N = 25)	Placebo (N = 25)
**Local symptoms**					
Pain	All	10 (40.0)	6 (24.0)	15 (60.0)	1 (4.0)
	Severe	0 (0.0)	0 (0.0)	0 (0.0)	0 (0.0)
Redness	All	0 (0.0)	0 (0.0)	0 (0.0)	0 (0.0)
	Severe	0 (0.0)	0 (0.0)	0 (0.0)	0 (0.0)
Swelling	All	1 (4.0)	0 (0.0)	0 (0.0)	0 (0.0)
	Severe	0 (0.0)	0 (0.0)	0 (0.0)	0 (0.0)
**Systemic symptoms**					
Arthralgia	All	2 (8.0)	2 (8.0)	0 (0.0)	0 (0.0)
	Severe	0 (0.0)	0 (0.0)	0 (0.0)	0 (0.0)
Fatigue	All	7 (28.0)	3 (12.0)	6 (24.0)	4 (16.0)
	Severe	0 (0.0)	0 (0.0)	0 (0.0)	0 (0.0)
Fever	All	0 (0.0)	0 (0.0)	1 (4.0)	0 (0.0)
	Severe	0 (0.0)	0 (0.0)	0 (0.0)	0 (0.0)
Headache	All	4 (16.0)	3 (12.0)	2 (8.0)	5 (20.0)
	Severe	0 (0.0)	0 (0.0)	0 (0.0)	0 (0.0)
Malaise	All	2 (8.0)	4 (16.0)	0 (0.0)	1 (4.0)
	Severe	0 (0.0)	0 (0.0)	0 (0.0)	0 (0.0)
Myalgia	All	2 (8.0)	2 (8.0)	1 (4.0)	2 (8.0)
	Severe	0 (0.0)	0 (0.0)	0 (0.0)	0 (0.0)
**Unsolicited adverse events**					
All		7 (28.0)	6 (24.0)	11 (44.0)	11 (44.0)
Severe		1 (4.0)	0 (0.0)	0 (0.0)	1 (4.0)
Related to the IP		0 (0.0)	1 (4.0)	2 (8.0)	0 (0.0)
Related to the IP and severe		0 (0.0)	0 (0.0)	0 (0.0)	0 (0.0)
**Serious adverse events**					
All		3 (12.0)	1 (4.0)	1 (4.0)	0 (0.0)
Related to the IP		0 (0.0)	0 (0.0)	0 (0.0)	0 (0.0)

## Data Availability

The data sharing plan has been communicated in the Clinical Study Protocol (§ 10.8.3.1) which is provided in the Supplementary Materials. The results have been published on the EudraCT website, as well as on the ClinicalTrials.gov website.

## Supplementary Materials

The following supporting information can be downloaded at: https://www.mdpi.com/article/10.3390/vaccines12121391/s1, Supplementary S1: Inclusion and exclusion criteria; Supplementary S2: Immunoassays [44]; Supplementary S3: Statistical methods; Supplementary S4: Raw immunogenicity data; Supplementary S5: Influence of sex and age on the baseline immunological readout values; Supplementary S6.1: Effect of the three dose levels (180 µg, 300 µg and 480 µg) of OVX836 on the difference (Day 8–Day 1) in nucleoprotein (NP)-specific interferon gamma (IFNγ) spot forming cells (SFCs) per 10^6^ peripheral blood mononuclear cells (PBMCs) (Panel A) and the percentage of CD4^+^ T-cells expressing at least IFNγ (Panel B) in the two age cohorts; Supplementary S6.2: Effect of the three dose levels (180 µg, 300 µg and 480 µg) of OVX836 on the difference (Day 8–Day 1) in the number of nucleoprotein (NP)-specific interferon gamma (IFNγ) spot forming cells (SFCs) per 10^6^ peripheral blood mononuclear cells (PBMCs) (Panel A) and in the % of CD4^+^ T-cells expressing at least IFNγ (Panel B), in female subjects as a function of age; Supplementary S6.3: Effect of the three dose levels (180 µg, 300 µg and 480 µg) of OVX836 on the difference (Day 8–Day 1) in the number of nucleoprotein (NP)-specific interferon gamma (IFNγ) spot forming cells (SFCs) per 10^6^ peripheral blood mononuclear cells (PBMCs) (Panel A) and in the % of CD4^+^ T-cells expressing at least IFNγ (Panel B), in male subjects as a function of age; Supplementary S7: Effect of the three dose levels (180 µg, 300 µg and 480 µg) of OVX836 and placebo on the Day 29/Day 1 anti-nucleoprotein (NP) immunoglobulin G (IgG) geometric mean ratios in the two age cohorts, displayed by sex (males on the left and females on the right); Supplementary S8: Analyses of the effects of baseline values, age category and sex on the immunological responses to OVX836, with pooled data from the three dose levels, using ANCOVA models.
