# Supplementary material for: Evaluation of Safety, Immunogenicity and Cross-Reactive Immunity of OVX836, a Nucleoprotein-Based Universal Influenza Vaccine, in Older Adults
Source: Vaccines (Basel). 2024 Dec 11;12(12):1391. doi: 10.3390/vaccines12121391 (PMC11728545; doi:10.3390/vaccines12121391)
Supplement: Supplementary file 1 [file vaccines-12-01391-s001.zip › Supplementary S1.pdf]

Supplementary S1: Inclusion and exclusion criteria

Inclusion criteria:

1. Written informed consent.
2. Healthy male or female subjects, as determined by medical history and medical examination.
3. Aged 65 years and older.
4. Subject who has fully been vaccinated with licensed SARS-CoV-2 (COVID-19) vaccine(s) according to national recommendations.
5. Reliable and willing to make themselves available for the duration of the study, and willing and able to follow study procedures.
6. Ability for completing a paper diary.

Exclusion criteria:

1. Subjects with a body mass index (BMI) $\leq$19 kg/m^2 or $\geq$35 kg/m^2 on the day of vaccination.
2. Any known or suspected immunodeficient conditions.
3. Past or current history of significant autoimmune diseases, as judged by the Investigator.
4. Current history of uncontrolled medical illness such as diabetes, hypertension, heart, renal or hepatic diseases.
5. Known or suspected infection with human immunodeficiency virus (HIV), hepatitis C virus (HCV), or hepatitis B virus (HBV).
6. Having received another vaccination within 3 months prior to the day of study vaccination for live attenuated vaccines, or within 1 month prior to the day of study vaccination for inactivated vaccines, except COVID-19 vaccine.
7. Planning to receive other vaccines during the first 28 days following the study vaccine administration, except COVID-19 vaccine.
8. Administration of any investigational or non-registered drug or vaccine within 3 months prior to the administration of study vaccines, or planned administration of any such product during the whole study period.
9. History of receiving blood, blood components or immunoglobulins within 3 months prior to the day of vaccination, or planned to receive such product during the whole study period.
10. Presence of an acute febrile illness on the day of vaccination (oral temperature >38.0°C, temporary exclusion criterion).
11. Past or current history of any progressive or severe neurological disorder, seizure disorder or Guillain-Barré syndrome.
12. Behavioural or cognitive impairment, or psychiatric disease that, in the opinion of the Investigator, may interfere with the subject's ability to participate in the study.
13. Past (stopped less than 6 months before enrolment) or current history of alcohol or drug abuse, or current smoking habit above 10 cigarettes per day, or current vaping.

14. Treatment that can affect immune response such as systemic or high dose inhaled corticosteroids (>800μg/day beclomethasone or equivalent; occasional inhaled corticosteroids for asthma therapy are allowed), radiation treatment, cytotoxic drugs, or current or recent (within 30 days before study entry) chronic or prolonged (>10 days) use of systemic non-steroidal anti-inflammatory drugs, interferon, immunomodulators, allergy shots, as judged by the Investigator.
15. History of severe allergic reactions and/or anaphylaxis, or serious adverse reactions to vaccines or allergy to kanamycin.
16. Any contraindication to IM administration, as judged by the Investigator.
17. Individuals with history of any illness that, in the opinion of the Investigator, might interfere with the results of the study or pose additional risk to the subjects due to participation in the study.
18. Sponsor employees or Investigator site personnel directly affiliated with this study, and their immediate families. Immediate family is defined as a spouse, parent, child or sibling, whether biological or legally adopted, including children of newly composed families.
19. Previous administration with OVX836 candidate vaccine.
20. Having received a COVID-19 vaccination within 2 weeks prior to the day of study vaccination.
21. Planning to receive COVID-19 vaccine during the first week (within 7 days) following the study vaccine administration. An interval of preferably 14 days is recommended. If for scheduling reasons, COVID-19 vaccine has to be given on Day 8, the vaccination should be administered after completion of the study procedures.
