# Supplementary material for: Evaluation of Safety, Immunogenicity and Cross-Reactive Immunity of OVX836, a Nucleoprotein-Based Universal Influenza Vaccine, in Older Adults
Source: Vaccines (Basel). 2024 Dec 11;12(12):1391. doi: 10.3390/vaccines12121391 (PMC11728545; doi:10.3390/vaccines12121391)
Supplement: Supplementary file 1 [file vaccines-12-01391-s001.zip › Supplementary S2.pdf]

Supplementary S2: Immunoassays

ELISPOT Assay for peripheral blood mononuclear cells (PBMCs)

The Enzyme-Linked Immunospot Assay (ELISPOT) method was used to measure the numbers of PBMCs that secreted IFNγ upon in vitro stimulation with nucleoprotein (NP) (NP-specific IFNγ SFCs).

After thawing, PBMCs were resuspended in culture medium and rested overnight. A total of $2x10^5$ PBMCs per well were incubated in triplicate wells for 24 hours with an NP peptide pool (>80% purity) at a final concentration of 4µg per peptide/mL. The NP peptide pool consisted of 122 overlapping 15-mer peptides spanning the amino-acid sequence of the NP from influenza A/WSN/33 (H1N1) strain, with overlaps of 11 amino acids. As a negative control, cells were incubated under the same conditions without the peptide pool (culture medium + 0.3% dimethyl-sulfoxide [DMSO]). The average SFCs from the negative control were subtracted from the average SFCs of the test wells, and results were reported as SFCs per million PBMCs for each clinical sample.

The ELISPOT assay was qualified using quality control (QC) samples. The method was specific. The Limit of Detection (LOD) was established at 29 SFC/million PBMC, with an estimated lower limit of quantification (LLOQ) of 82 SFC/million PBMC. The estimated upper limit of quantification (ULOQ) was 3743 SFC/million PBMC, based on an anti-CD3 positive control, as no samples exhibiting a high NP signal were available for the assay qualification. The method exhibited acceptable within-run and between-run precision for samples exceeding the LLOQ, with a coefficient of variation [CV] within-run of <30% and a CV between-run of <50% over at least 3 days, with 3 replicates of triplicate wells per day.

One internal QC sample was tested on each plate in parallel with the evaluation of clinical samples to validate the plate according to predefined criteria. If criteria were not met, the plate was considered invalid, and a retest was performed. Data below the LOD for clinical samples were reported as 15 SFC/million PBMC (1/2 LOD) for statistical analyses.

Cross-reactivity of the NP-specific responses evaluated by IFNγ ELISPOT

The aim of this evaluation was to characterize the reactivity of the specific cellular immune response induced by OVX836 against the NP from heterologous Influenza virus A and B strains. Two evaluations were conducted sequentially in samples from subjects aged 18-55 years and in those aged 65 years and older:

- **First evaluation**: This involved cell samples from 35 subjects (26 females and 9 males) aged 18-55 years who had received the OVX836 vaccine at either the 300 µg or 480 µg dose level. Subjects in the 300µg group were identified as good responders to the vaccination, exhibiting a low baseline signal and a Day 8/Day 1 IFNγ ELISPOT ratio of ≥2.5. Subjects in the 480µg group were selected randomly.
- **Second evaluation**: This involved cell samples from 24 subjects (18 females and 6 males) aged 65 to 81 years, who had received the OVX836 vaccine at dose levels of 180 µg, 300

µg or 480 µg (8 subjects per dose-level) and exhibited a D8/D1 ratio >2 based on IFNγ ELISPOT results.

The selected PBMC samples were stimulated with NP peptide pools from H1N1-A/WSN/1933 (homologous to OVX836) and heterologous strains pH1N1-A/California/04/2009, H3N2-A/Kansas/14/2017, H5N1-A/Indonesia/5/2005 and B/Colorado/06/2017. The IFNγ ELISPOT assay was used to enumerate specific precursor cells capable of producing IFNγ upon stimulation with the relevant antigens and to compare the responses.

The degrees of similarity of the amino-acid sequences of the NP used in this exploratory study versus the NP from H1N1-A/WSN/1933 strain included in OVX836 are presented in the table below.

Sequence similarity (% identities and % similarities [positives]) of the nucleoprotein (NP) of Influenza A or B strains to the NP sequence of Influenza H1N1-A/WSN/1933 from OVX836. Sequence alignment Blast on NIH website (https://blast.ncbi.nlm.nih.gov/Blast.cgi)

	**% Identities** “Two sequences have the same residues at the same position in the alignment”	**% Positives** “Non identical substitutions that receive a positive score, positive indicates a conservative substitution or substitutions that are often observed in related proteins”
**NP pH1N1-A/California/04/2009**	92%	97%
**NP H3N2-A/Kansas/14/2017**	91%	96%
**NP H5N1-A/Indonesia/5/2005**	94%	98%
**NP Victoria B/Colorado/06/2017**	37%	57%

Flow cytometry – Intracellular staining (ICS) for PBMC

An adaptation of the method described by Moris et al. (2011)[1] was used to measure NP-specific $CD4^+$ and $CD8^+$ T-cells positive for interleukin 2 (IL-2), tumour necrosis factor alpha (TNFα) and interferon gamma (IFNγ).

After thawing, the PBMC were washed with culture medium and incubated in vitro with the relevant antigen or corresponding medium in the presence of costimulatory antibodies to CD28 and CD49d for two hours. Brefeldin A, a cytokine secretion blocker, was then added for an overnight incubation, which inhibited cytokine secretion and allowed for its accumulation in the expressing cells. The following day, the cells were stained with fluorochrome-conjugated antibodies targeting phenotypic markers (CD3, CD4 and CD8) as well as activation/cytokine markers (IFNγ, IL-2 and TNFα). The stained cell samples were subsequently analysed using flow cytometry.

The relevant antigen was the same peptide pool used in ELISPOT assay and consisted of 122 overlapping 15-mer peptides spanning the amino-acid sequence of the NP from influenza A/WSN/33 (H1N1) strain, with overlaps of 11 amino acids. The concentration for stimulation

[1] Moris, P., van der Most, R., Leroux-Roels, I., et al. (2011) H5N1 influenza vaccine formulated with AS03 A induces strong cross-reactive and polyfunctional CD4 T-cell responses. J. Clin. Immunol. 31(3), 443-454

was 4µg per peptide per mL, with a negative control (including 0.3% DMSO) tested for each sample.

NP-specific $CD4^+$ and $CD8^+$ T Cells were determined by cytometry as the $CD3^+CD4^+$ and $CD3^+CD8^+$ events expressing one or more cytokines among IFNγ, IL-2 and TNFα following *in vitro* stimulation with relevant antigen. The corresponding signal from the same sample after *in vitro* stimulation with medium + DMSO (blank) was subtracted. The ICS results were reported as frequencies (%) of NP-specific $CD4^+$ and $CD8^+$ T-cells relative to the total $CD4^+$ and $CD8^+$ T cell populations, respectively. Final results below 0.0001% were set at 0.0001%.

Anti-NP serum immunoglobulin G (IgG) antibody

The method used to measure anti-NP serum IgG antibody was an indirect Enzyme-Linked Immunosorbent Assay (ELISA), which included the following steps: 1) coating the wells of the plate with NP (Osivax R&D batch), 2) blocking non-specific sites using bovine serum albumin, 3) incubating the clinical samples after two-fold serial dilutions (starting at a dilution of 1:800), 4) adding a secondary anti-human IgG antibody conjugated to Horse Radish Peroxidase (HRP), and 5) revealing the signal using 3,3',5,5'-tetramethylbenzidine (TMB) substrate. The anti-NP IgG titre for a serum sample was defined as the highest dilution that produced an optical density greater than two times the signal of the negative control tested on each plate.

The method was qualified using various positive (QC+) and negative (QC-) serum controls. It demonstrated specificity, selectivity and acceptable within-run precision (n=6, CV<20% for optical densities; QC+ titre: 6400 ± 1 dilution) and between-run precision (CV <30% on optical densities; QC+ titre: 6400 ± 1 dilution, n=3 tests on 3 different days with 2 operators). The stability of the QC samples was assessed after three freeze-thaw cycles and after 5 months at -20°C. QC samples were used in parallel with clinical samples to validate each plate according to predefined criteria; if the criteria were not met, the plate was deemed invalid, and a retest was performed.

Anti-OVX313 IgG and anti-hC4BP oligomerization domain IgG

An indirect ELISA was used to titrate serum anti-OVX313 or anti-hC4BP oligomerization domain IgG antibodies. It involved the following steps: 1) coating the wells of the plate with OVX313 or hC4BP oligomerization domain (Osivax R&D batches), 2) blocking non-specific sites with bovine serum albumin, 3) incubating the clinical samples after two-fold serial dilutions (starting at a dilution of 1:12.5), 4) adding a secondary anti-human IgG antibody conjugated to Horse Radish Peroxidase (HRP), and 5) revealing the signal using 3,3',5,5'-TMB substrate.

The anti-OVX313 or anti-hC4BP oligomerization domain IgG titre post-OVX836 vaccination for a serum sample was defined as the highest dilution that produced an optical density greater than two times the baseline optical density at. A value of 6.25 was reported when the titre was less than 12.5 (the first dilution applied).
