# Supplementary material for: Evaluation of Safety, Immunogenicity and Cross-Reactive Immunity of OVX836, a Nucleoprotein-Based Universal Influenza Vaccine, in Older Adults
Source: Vaccines (Basel). 2024 Dec 11;12(12):1391. doi: 10.3390/vaccines12121391 (PMC11728545; doi:10.3390/vaccines12121391)
Supplement: Supplementary file 1 [file vaccines-12-01391-s001.zip › Supplementary S3.pdf]

Supplementary S3: Statistical methods

Descriptive statistics included the arithmetic mean or geometric mean, standard deviation, 95% confidence interval, minimum and maximum for continuous variables, and absolute number and percentage for discrete variables.

For all inferential analyses, a significance level of α=5% was applied. No corrections for multiplicity were made, as the statistical tests were exploratory in nature, and results should therefore be interpreted with caution.

In the immunogenicity analyses of subjects aged 65 years and older, intergroup comparisons were performed using Kruskal-Wallis tests, followed by Mann-Whitney tests when significant. Intragroup comparisons were performed using Friedman tests, followed by Wilcoxon signed-rank tests when significant.

Cross-reactivity responses, defined as differences in the NP-specific responses between Day 8 and Day 1 for homologous and heterologous strains were assessed for correlation using linear regression. This included the determination of the slope (beta coefficient with associated p value), Spearman's coefficient of correlation (r), and coefficient of determination (R^2).

Analyses of variance (ANOVA) or independent Student's t tests have been used to assess the influence of baseline values on the immune response to OVX836. This was evaluated in terms of difference between Day 8 and Day 1 (pre-vaccination) in the number of NP-specific IFNγ SFCs per million PBMCs, differences in the percentage of NP-specific $CD4^+$ T-cells positive for at least IFNγ , and Day 8/Day 1 and Day 29/Day 1 ratios for anti-NP IgG.

Two-way ANOVA was used to assess the impact of sex, age category and their interaction on baseline immunological values.

Immunological responses to the three dose levels of OVX836 (180 µg, 300 µg and 480 µg) and the pooled OVX836 group (combining all doses) were analysed using analysis of covariance (ANCOVA) models, accounting for sex and age category as fixed factors, and baseline values as covariates.
