# Supplementary material for: Evaluation of Safety, Immunogenicity and Cross-Reactive Immunity of OVX836, a Nucleoprotein-Based Universal Influenza Vaccine, in Older Adults
Source: Vaccines (Basel). 2024 Dec 11;12(12):1391. doi: 10.3390/vaccines12121391 (PMC11728545; doi:10.3390/vaccines12121391)
Supplement: Supplementary file 1 [file vaccines-12-01391-s001.zip › Supplementary S4.pdf]

Supplementary S4: Raw immunogenicity data

Number of NP-specific IFNγ spot forming T-cells (SFC) per million PBMCs at Day 1 (vaccination day) and Day 8 in the four treatment groups

Visit	Statistics	Placebo	OVX836 (180µg)	OVX836 (300µg)	OVX836 (480µg)
Day 1	n	25	25	24	25
	Mean	81.9	86.5	64.7	57.8
	95% CI	51.6 , 112.2	11.1 , 162.0	35.7 , 93.6	31.0 , 84.5
	SD	73.4	182.8	68.5	64.8
	Median	65.0	35.0	42.5	43.0
	Minimum	15	15	15	15
	Maximum	348	938	313	330
Day 8	n	25	25	24	24
	Mean	82.7	235.2	219.6	167.6
	95% CI	47.7 , 117.7	108.2 , 362.3	103.1 , 336.1	109.1 , 226.1
	SD	84.9	307.8	275.9	138.5
	Median	57.0	155.0	147.0	155.5
	Minimum	15	15	15	15
	Maximum	337	1390	1037	518

Percentage of NP-specific CD4+ T-cells positive for at least IFNγ, or for IFNγ and IL-2 at baseline (Day 1) and Day 8, in the four treatment groups

Marker	Time	Treatment	N	Mean	SD	Median	LL 95% CI	UL 95% CI
At least IFNγ	Day 1	Placebo	25	0.024	0.017	0.022	0.017	0.031
		OVX836 180µg	25	0.018	0.021	0.014	0.009	0.026
		OVX836 300µg	24	0.022	0.016	0.017	0.015	0.029
		OVX836 480µg	25	0.014	0.013	0.010	0.008	0.019
	Day 8	Placebo	25	0.021	0.019	0.019	0.013	0.029
		OVX836 180µg	25	0.088	0.062	0.081	0.062	0.114
		OVX836 300µg	24	0.116	0.102	0.083	0.072	0.159
		OVX836 480µg	24	0.085	0.055	0.078	0.062	0.109
IFNγ and IL-2	Day 1	Placebo	25	0.004	0.004	0.002	0.002	0.005
		OVX836 180µg	25	0.003	0.004	0.001	0.001	0.004
		OVX836 300µg	24	0.004	0.004	0.003	0.002	0.006
		OVX836 480µg	25	0.003	0.002	0.003	0.002	0.004
	Day 8	Placebo	25	0.004	0.006	0.002	0.002	0.007
		OVX836 180µg	25	0.028	0.019	0.026	0.020	0.035
		OVX836 300µg	24	0.036	0.034	0.029	0.022	0.051
		OVX836 480µg	24	0.030	0.024	0.025	0.019	0.040

N = number of subjects with results available and valid in the PP-D8; SD = standard deviation; LL and UL 95%CI = lower and upper limit of the 95% confidence interval.

Percentage of NP-specific CD8+ T-cells positive for at least IFNγ, or for IFNγ and IL-2 at baseline (Day 1) and Day 8, in the four treatment groups

Marker	Time	Treatment	N	Mean	SD	Median	LL 95% CI	UL 95% CI
**At least IFNγ**	**Day 1**	**Placebo**	24	0.110	0.172	0.047	0.037	0.182
		**OVX836 180µg**	23	0.095	0.199	0.038	0.009	0.181
		**OVX836 300µg**	23	0.052	0.095	0.035	0.011	0.093
		**OVX836 480µg**	24	0.097	0.153	0.039	0.032	0.161
	**Day 8**	**Placebo**	24	0.101	0.114	0.053	0.053	0.149
		**OVX836 180µg**	23	0.122	0.297	0.040	-0.000	0.245
		**OVX836 300µg**	23	0.120	0.224	0.027	0.025	0.214
		**OVX836 480µg**	24	0.095	0.126	0.074	0.040	0.150
**IFNγ and IL-2**	**Day 1**	**Placebo**	24	0.016	0.023	0.008	0.006	0.025
		**OVX836 180µg**	23	0.011	0.028	0.004	-0.001	0.023
		**OVX836 300µg**	23	0.010	0.021	0.000	0.001	0.020
		**OVX836 480µg**	24	0.011	0.019	0.002	0.003	0.019
	**Day 8**	**Placebo**	24	0.014	0.022	0.003	0.005	0.023
		**OVX836 180µg**	25	0.018	0.038	0.006	0.002	0.034
		**OVX836 300µg**	24	0.010	0.019	0.003	0.002	0.017
		**OVX836 480µg**	23	0.013	0.016	0.005	0.006	0.020

N = number of subjects with results available and valid in the PP-D8; SD = standard deviation; LL and UL 95%CI = lower and upper limit of the 95% confidence interval.

Anti-NP IgG titers at pre-injection baseline (Day 1), Day 8 and Day 29, in the four treatment groups

Time	Treatment	N	GMT	Median	Min	Max	LL 95% CI	UL 95% CI
**Day 1**	**Placebo**	25	5419	6400	1600	25600	4057	7238
	**OVX836 180µg**	25	6225	6400	1600	25600	4597	8430
	**OVX836 300µg**	24	5080	6400	1600	12800	3884	6643
	**OVX836 480µg**	25	6955	6400	800	102400	4447	10878
**Day 8**	**Placebo**	25	5127	6400	1600	51200	3776	6962
	**OVX836 180µg**	25	9973	12800	3200	25600	7909	12576
	**OVX836 300µg**	24	9589	12800	3200	25600	7307	12584
	**OVX836 480µg**	25	16890	25600	1600	51200	12015	23742
**Day 29**	**Placebo**	25	5127	6400	1600	51200	3776	6962
	**OVX836 180µg**	25	19401	25600	6400	102400	14404	26131
	**OVX836 300µg**	24	17086	12800	3200	102400	12109	24109
	**OVX836 480µg**	25	26320	25600	1600	102400	17070	40581

N = number of subjects with results available and valid in the PP-D8; GMT = geometric mean titer; Min= Minimum; Max = Maximum; LL and UL 95%CI = lower and upper limit of the 95% confidence interval.
