# Supplementary material for: Evaluation of Safety, Immunogenicity and Cross-Reactive Immunity of OVX836, a Nucleoprotein-Based Universal Influenza Vaccine, in Older Adults
Source: Vaccines (Basel). 2024 Dec 11;12(12):1391. doi: 10.3390/vaccines12121391 (PMC11728545; doi:10.3390/vaccines12121391)
Supplement: Supplementary file 1 [file vaccines-12-01391-s001.zip › Supplementary S5.pdf]

Supplementary S5: Influence of sex and age on the baseline immunological readout values

The baseline number of NP-specific IFNγ SFCs per 10^6 PBMCs, as analysed by ELISPOT, was significantly higher in males compared to females ($p=0.035$), and in subjects aged 18-55 years compared to those aged ≥65 years ($p=0.006$), with no interaction between age and sex.

The baseline percentage of $CD4^+$ T-cells expressing at least IFNγ was significantly higher in subjects aged 18-55 years compared to those aged ≥65 years ($p=0.005$), with no significant effect of sex, and no interaction between age and sex.

The baseline percentage of $CD8^+$ T-cells expressing at least IFNγ was significantly higher in males compared to females ($p=0.026$), with no effect of age category, and no interaction between age and sex.

Baseline anti-NP IgG titres were significantly lower in subjects aged 18-55 years-old compared to those aged ≥65 years ($p<0.0001$), with no effect of sex, and no interaction between age and sex factors.

Baseline (pre-vaccination) immunological values, including the number of NP-specific IFNγ SFCs per million PBMCs determined by ELISPOT, the percentage of $CD4^+$ and $CD8^+$ T-cells expressing at least IFNγ determined by intracellular staining, and anti-NP IgG geometric mean titres [GMT]) determined by ELISA, as a function of sex and age category. Results are presented as means (arithmetic or geometric), standard deviations (SD) and medians

		18-55 years-old Males	18-55 years-old Females	18-55 years-old Total	≥65 years-old Males	≥65 years-old Females	≥65 years-old Total
Number of NP-specific IFNγ SFCs per 10^6 PBMCs	N	36	94	130	50	49	99
	Mean	126	95	104	87	59	73
	SD	109	82	91	145	46	109
	Median	95	73	79	45	43	43
% of CD4 T-cells expressing at least IFNγ	N	36	94	130	50	49	99
	Mean	0.0302	0.0244	0.0260	0.0198	0.0190	0.0194
	SD	0.0291	0.0176	0.0214	0.0199	0.0145	0.0173
	Median	0.0245	0.0229	0.0233	0.0139	0.0145	0.0141
% of CD8 T-cells expressing at least IFNγ	N	36	94	130	47	47	94
	Mean	0.1013	0.0672	0.0766	0.1098	0.0676	0.0887
	SD	0.1127	0.0677	0.0836	0.2078	0.0803	0.1581
	Median	0.078	0.0480	0.0507	0.0326	0.0411	0.0385
Anti-NP IgG	N	36	94	130	50	49	99
	GMT	4344	3965	4070	10144	7069	8622
	Median	3200	3200	3200	6400	6400	6400
