# Supplementary material for: Evaluation of Safety, Immunogenicity and Cross-Reactive Immunity of OVX836, a Nucleoprotein-Based Universal Influenza Vaccine, in Older Adults
Source: Vaccines (Basel). 2024 Dec 11;12(12):1391. doi: 10.3390/vaccines12121391 (PMC11728545; doi:10.3390/vaccines12121391)

Supplementary S6.1: Effect of the three dose levels (180 µg, 300 µg and 480 µg) of OVX836 on the difference (Day 8 – Day 1) in nucleoprotein (NP)-specific interferon gamma (IFNγ) spot forming cells (SFCs) per 10^6 peripheral blood mononuclear cells (PBMCs) (Panel A) and the percentage of $CD4^+$ T-cells expressing at least IFNγ (Panel B) in the two age cohorts. Results are presented as medians with individual data points. *p<0.05; **p<0.01.

Panel A

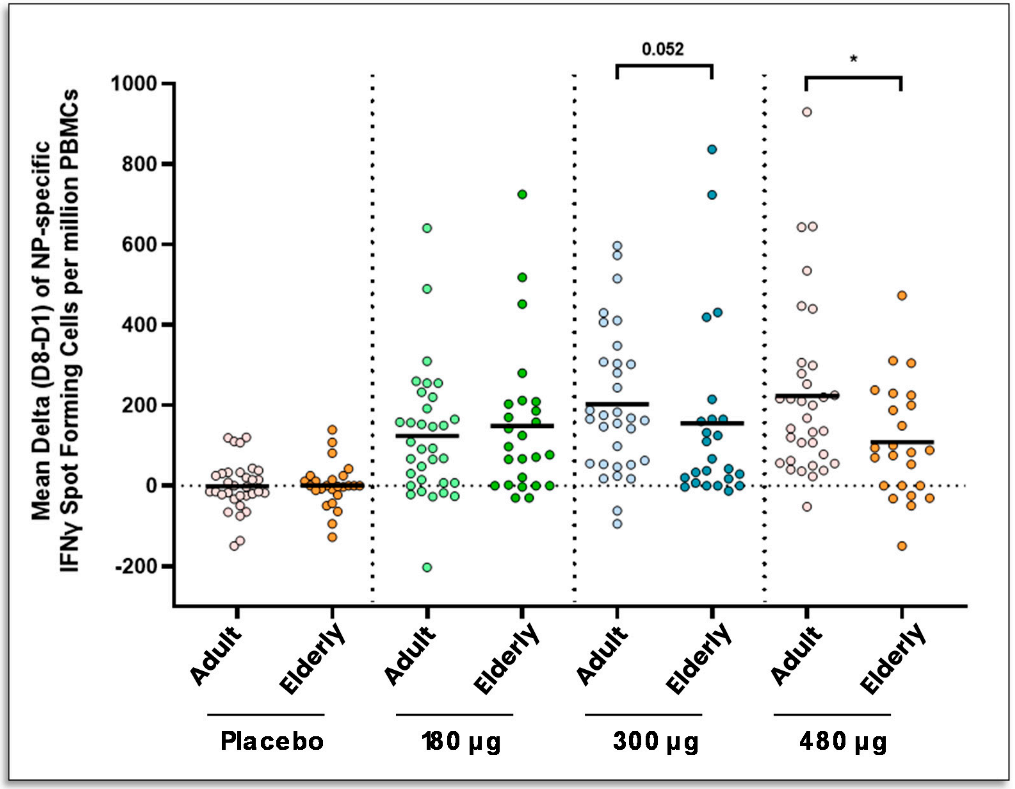

Panel B

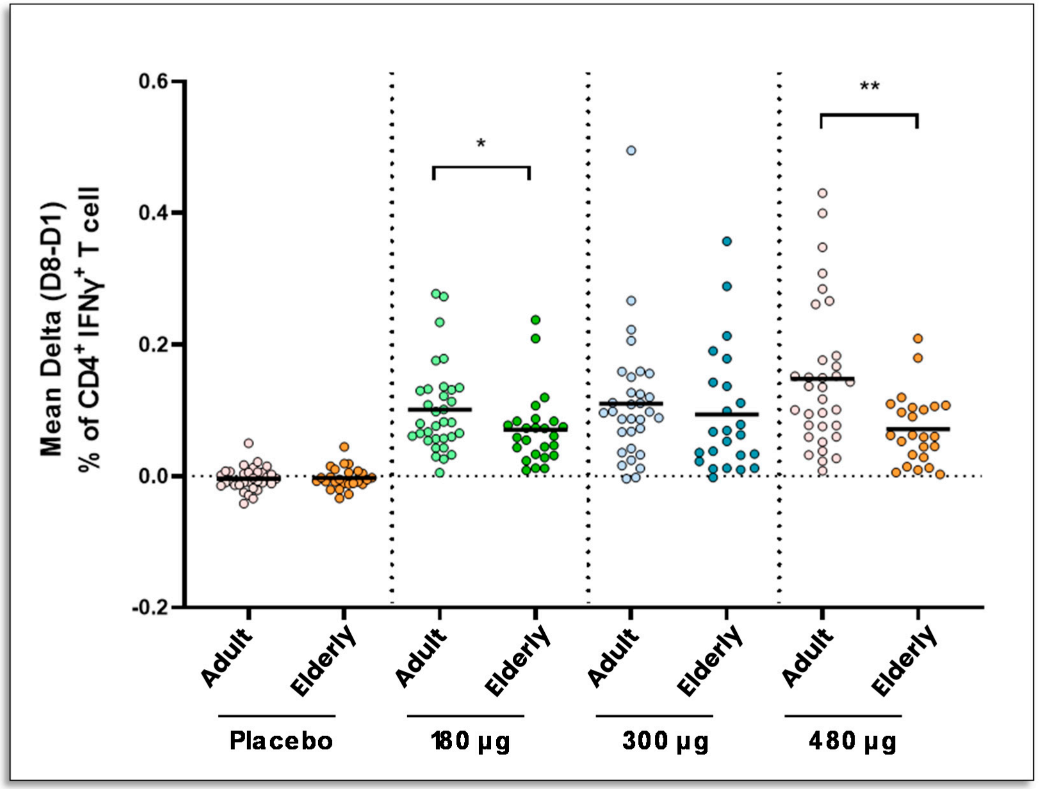

Supplementary S6.2: Effect of the three dose levels (180 µg, 300 µg and 480 µg) of OVX836 on the difference (Day 8 – Day 1) in the number of nucleoprotein (NP)-specific interferon gamma (IFNγ) spot forming cells (SFCs) per 10^6 peripheral blood mononuclear cells (PBMCs) (Panel A) and in the % of $CD4^+$ T-cells expressing at least IFNγ (Panel B), in female subjects as a function of age. Results are shown as medians ± 95% confidence interval and individual data. *p<0.05; **p<0.01; ***p<0.001; ****p<0.0001.

Panel A

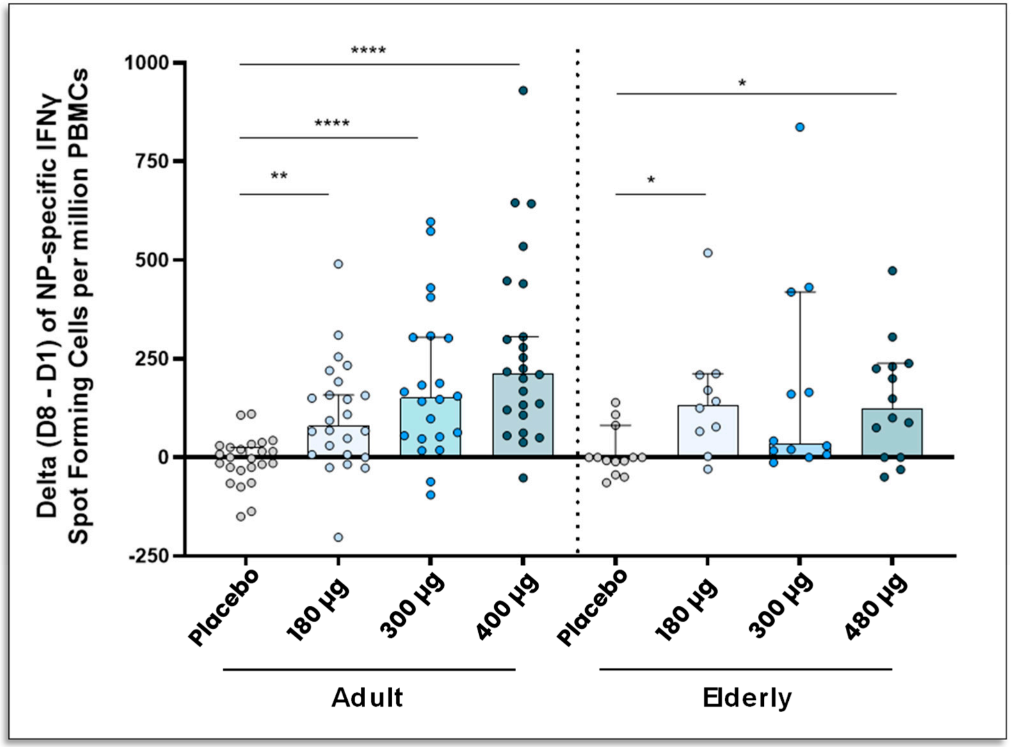

Panel B

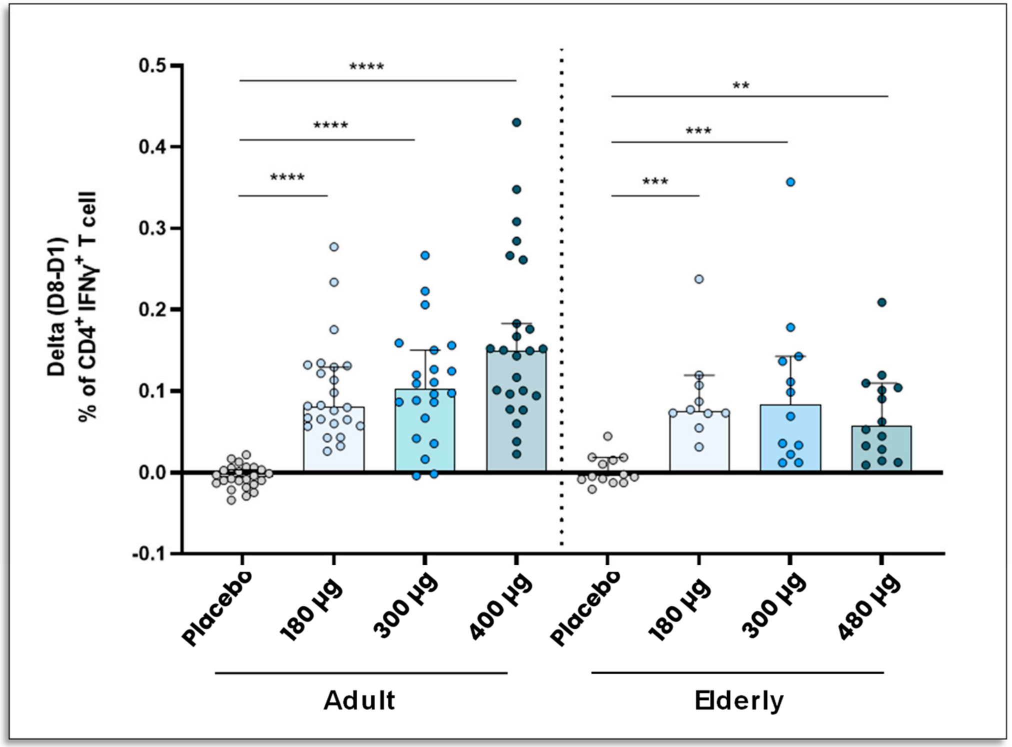

Supplementary S6.3: Effect of the three dose levels (180 µg, 300 µg and 480 µg) of OVX836 on the difference (Day 8 – Day 1) in the number of nucleoprotein (NP)-specific interferon gamma (IFNγ) spot forming cells (SFCs) per 10^6 peripheral blood mononuclear cells (PBMCs) (Panel A) and in the % of $CD4^+$ T-cells expressing at least IFNγ (Panel B), in male subjects as a function of age. Results are shown as medians ± 95% confidence interval and individual data. *p<0.05; **p<0.01; ***p<0.001.

Panel A

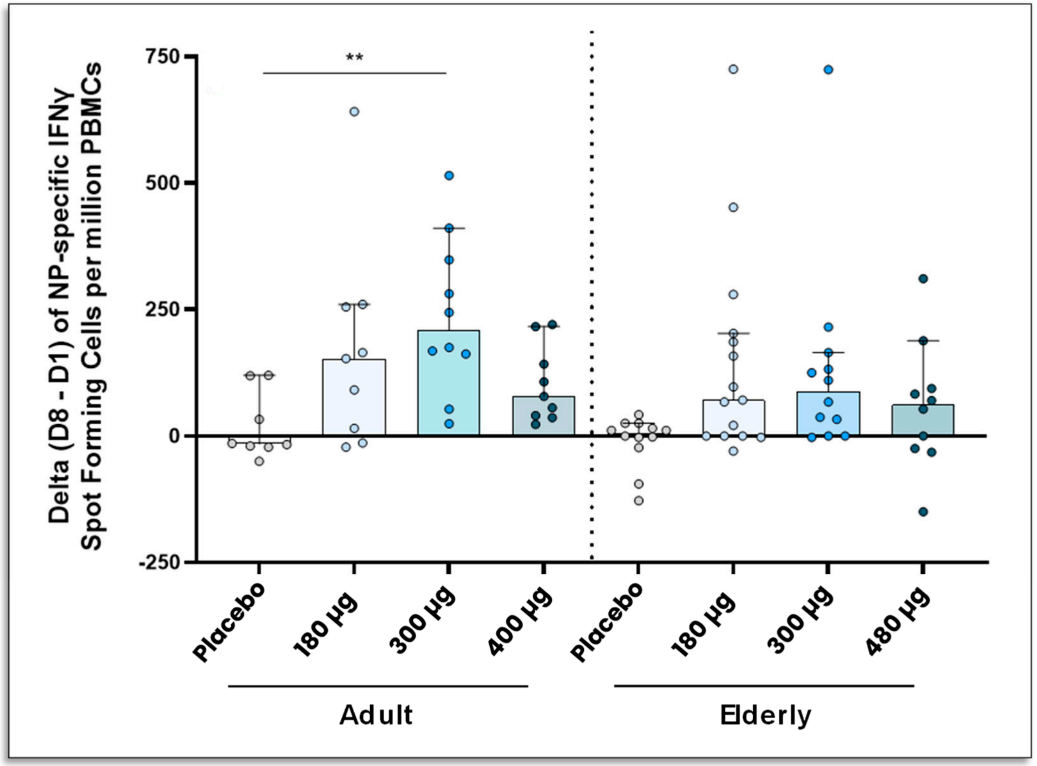

Panel B

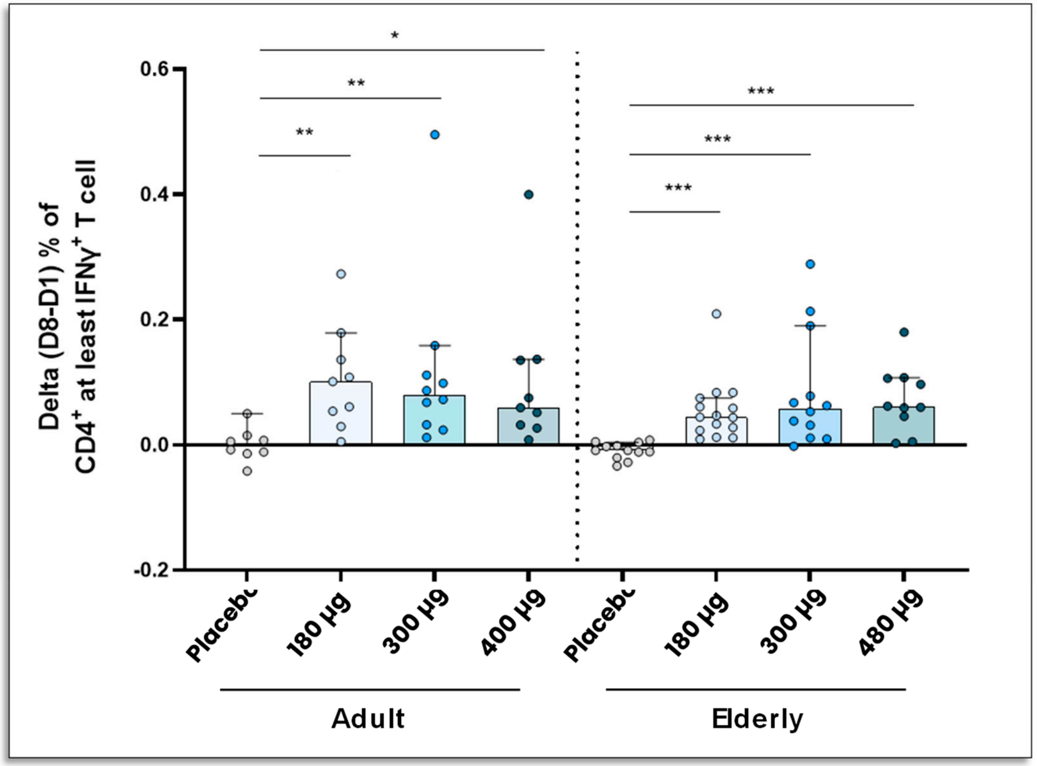

Supplement: Supplementary file 1 [file vaccines-12-01391-s001.zip › Supplementary S6.1 to 6.3.pdf]
