# Supplementary material for: Evaluation of Safety, Immunogenicity and Cross-Reactive Immunity of OVX836, a Nucleoprotein-Based Universal Influenza Vaccine, in Older Adults
Source: Vaccines (Basel). 2024 Dec 11;12(12):1391. doi: 10.3390/vaccines12121391 (PMC11728545; doi:10.3390/vaccines12121391)

Supplementary S7: Effect of the three dose levels (180 µg, 300 µg and 480 µg) of OVX836 and placebo on the Day 29/Day 1 anti-nucleoprotein (NP) immunoglobulin G (IgG) geometric mean ratios in the two age cohorts, displayed by sex (males on the left and females on the right). Results are presented as geometric mean ratio + standard deviation.

Males

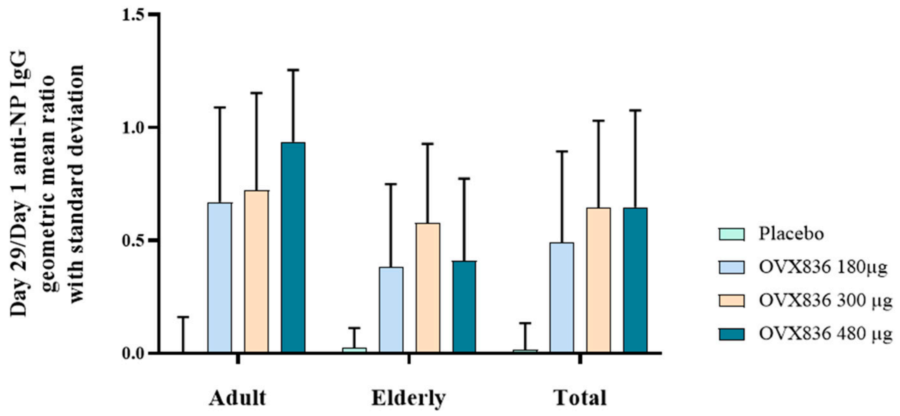

Females

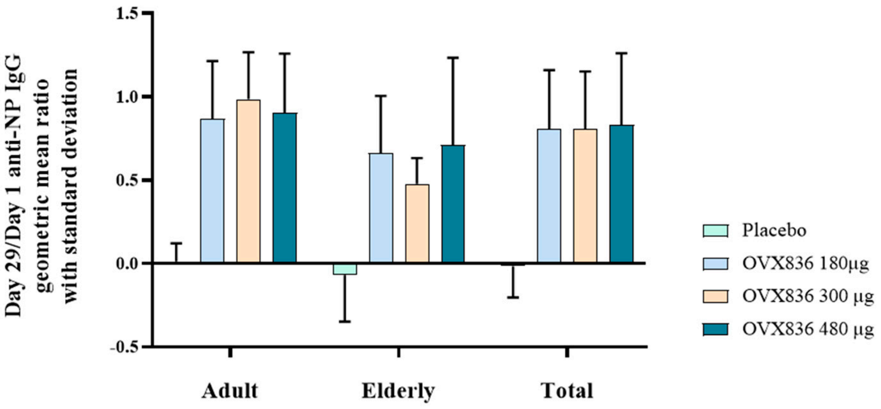

Supplement: Supplementary file 1 [file vaccines-12-01391-s001.zip › Supplementary S7.pdf]
