# Supplementary material for: Evaluation of Safety, Immunogenicity and Cross-Reactive Immunity of OVX836, a Nucleoprotein-Based Universal Influenza Vaccine, in Older Adults
Source: Vaccines (Basel). 2024 Dec 11;12(12):1391. doi: 10.3390/vaccines12121391 (PMC11728545; doi:10.3390/vaccines12121391)
Supplement: Supplementary file 1 [file vaccines-12-01391-s001.zip › Supplementary S8.pdf]

Supplementary S8: Analyses of the effects of baseline values, age category and sex on the immunological responses to OVX836, with pooled data from the three dose levels, using ANCOVA models

Number of NP-specific IFNγ SFCs per 10^6 PBMCs

Between-Subjects Factors

		Value Label	N
**Adult or elderly**	**1**	Adult	98
	**2**	Elderly	73
**Sex**	**1**	Male	65
	**2**	Female	106

Descriptive Statistics

Dependent Variable: Elispost differences D8 - D1

Adult or elderly	Sex	Mean	Std. Deviation	N
**Adult**	**Male**	172.9643	159.53625	28
	**Female**	186.7571	200.28548	70
	**Total**	182.8163	188.83497	98
**Elderly**	**Male**	119.5676	184.82718	37
	**Female**	155.7500	188.50682	36
	**Total**	137.4110	186.24229	73
**Total**	**Male**	142.5692	175.10898	65
	**Female**	176.2264	196.01896	106
	**Total**	163.4327	188.53219	171

Tests of Between-Subjects Effects

Dependent Variable: Elispost differences D8 - D1

Source	Type III Sum of Squares	df	Mean Square	F	Sig.
**Corrected Model**	500706.382[a]	4	125176.596	3.750	.006
**Intercept**	1282738.502	1	1282738.502	38.423	<.001
**ElispotD1**	386762.073	1	386762.073	11.585	<.001
**Agecategory**	27422.474	1	27422.474	.821	.366
**Sex**	47926.311	1	47926.311	1.436	.233
**Agecategory * Sex**	12205.167	1	12205.167	.366	.546
**Error**	5541839.594	166	33384.576		
**Total**	10610001.000	171			
**Corrected Total**	6042545.977	170			

a. R Squared = .083 (Adjusted R Squared = .061)

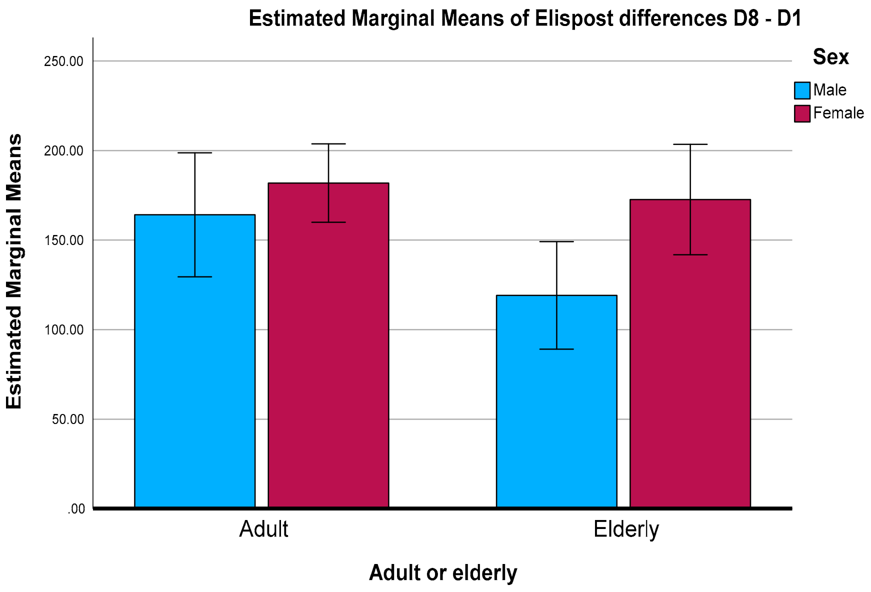

Covariates appearing in the model are evaluated at the following values: ELISPOT IFNg (NP-specific) D1 = 87.585

Error bars: +/- 1 SE

% of $CD4^+$ T-cells

Between-Subjects Factors

		Value Label	N
**Adult or elderly**	**1**	Adult	98
	**2**	Elderly	73
**Sex**	**1**	Male	65
	**2**	Female	106

Descriptive Statistics

Dependent Variable: CD4 at least IFNg D8 - D1

Adult or elderly	Sex	Mean	Std. Deviation	N
**Adult**	**Male**	.1081	.11365	28
	**Female**	.1242	.08448	70
	**Total**	.1196	.09341	98
**Elderly**	**Male**	.0699	.06685	37
	**Female**	.0869	.07207	36
	**Total**	.0783	.06952	73
**Total**	**Male**	.0864	.09126	65
	**Female**	.1116	.08207	106
	**Total**	.1020	.08629	171

Tests of Between-Subjects Effects

Dependent Variable: CD4 at least IFNg D8 - D1

Source	Type III Sum of Squares	df	Mean Square	F	Sig.
**Corrected Model**	.168[a]	4	.042	6.352	<.001
**Intercept**	.371	1	.371	56.087	<.001
**spCD4atleastIFNg.1**	.086	1	.086	13.017	<.001
**Agecategory**	.028	1	.028	4.262	.041
**Sex**	.013	1	.013	1.974	.162
**Agecategory * Sex**	.000	1	.000	.067	.796
**Error**	1.098	166	.007		
**Total**	3.044	171			
**Corrected Total**	1.266	170			

a. R Squared = .133 (Adjusted R Squared = .112)

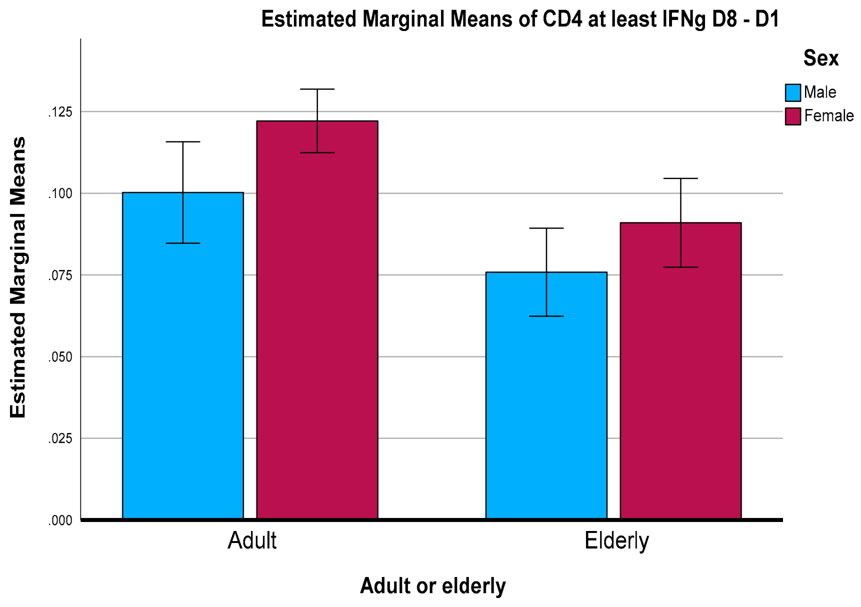

Covariates appearing in the model are evaluated at the following values: CD4+ at least IFNg D1 = .022353

Error bars: +/- 1 SE

Anti-NP IgG Day 29/Day 1 geometric mean ratio

Between-Subjects Factors

		Value Label	N
**Adult or elderly**	**1**	Adult	98
	**2**	Elderly	74
**Sex**	**1**	Male	66
	**2**	Female	106

Descriptive Statistics

Dependent Variable: Log (titer D29/titer D1)

Adult or elderly	Sex	Mean	Std. Deviation	N
**Adult**	**Male**	.7741	.39657	28
	**Female**	.9160	.32990	70
	**Total**	.8754	.35404	98
**Elderly**	**Male**	.4515	.36198	38
	**Female**	.6188	.38714	36
	**Total**	.5329	.38126	74
**Total**	**Male**	.5884	.40708	66
	**Female**	.8151	.37614	106
	**Total**	.7281	.40260	172

Tests of Between-Subjects Effects

Dependent Variable: Log (titer D29/titer D1)

Source	Type III Sum of Squares	df	Mean Square	F	Sig.
**Corrected Model**	11.009[a]	4	2.752	27.509	<.001
**Intercept**	9.269	1	9.269	92.644	<.001
**LogAntiNPIgGD1**	5.142	1	5.142	51.393	<.001
**Agecategory**	.857	1	.857	8.569	.004
**Sex**	.515	1	.515	5.145	.025
**Agecategory * Sex**	.056	1	.056	.556	.457
**Error**	16.708	167	.100		
**Total**	118.892	172			
**Corrected Total**	27.717	171			

a. R Squared = .397 (Adjusted R Squared = .383)

Estimated Marginal Means of Log (titer D29/titer D1)

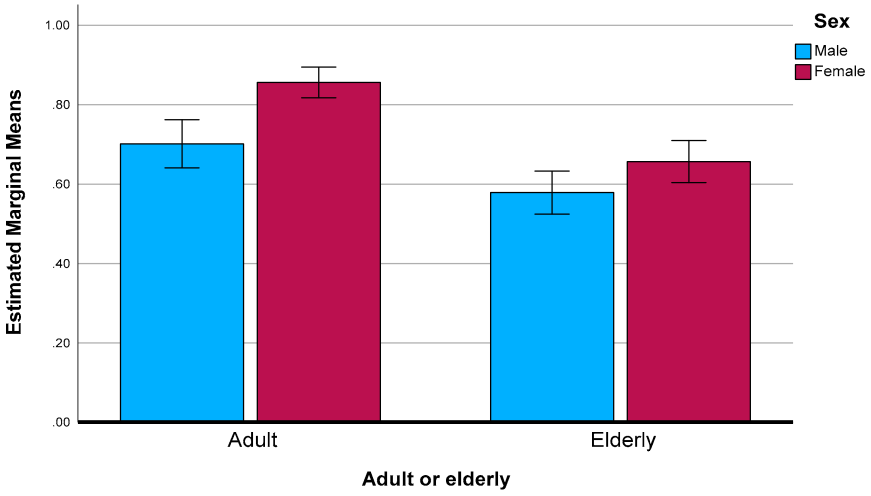

Covariates appearing in the model are evaluated at the following values: Log anti-NP IgG D1 = 3.6277

Error bars: +/- 1 SE
